# The Development and Optimization of Lipid-Based Self-Nanoemulsifying Drug Delivery Systems for the Intravenous Delivery of Propofol

**DOI:** 10.3390/molecules28031492

**Published:** 2023-02-03

**Authors:** Mohsin Kazi, Athba Alqahtani, Majed Alharbi, Ajaz Ahmad, Muhammad Delwar Hussain, Hani Alothaid, Mohammed S. Aldughaim

**Affiliations:** 1 Kayyali Chair for Pharmaceutical Industries, Department of Pharmaceutics, College of Pharmacy, King Saud University, P.O. Box 2457, Riyadh 11451, Saudi Arabia; 2 Research Center, King Fahad Medical City, Riyadh 11451, Saudi Arabia; 3 Clinical Pharmacy, College of Pharmacy, King Saud University, P.O. Box 2457, Riyadh 11451, Saudi Arabia; 4 Department of Pharmaceutical Sciences, School of Pharmacy, College of Health and Pharmacy, Husson University, Bangor, ME 04401, USA; 5 Department of Basic Sciences, Faculty of Applied Medical Sciences, Al-Baha University, Al Baha 65528, Saudi Arabia

**Keywords:** self-nanoemulsifying drug delivery systems, propofol, pharmacokinetics, sleeping disorder, intravenous delivery

## Abstract

Purpose: Propofol is a relatively short-acting potent anesthetic lipophilic drug used during short surgical procedures. Despite the success of propofol intravenous emulsions, drawbacks to such formulations include inherent emulsion instability, the lack of a safe vehicle to prevent sepsis, and concern regarding hyperlipidemia-related side effects. The aim of the current investigation was to develop a novel, lipid-based self-nanoemulsifying drug delivery system (SNEDDS) for propofol with improved stability and anesthetic activity for human use. Methods: A series of SNEDDS formulations were developed using naturally obtained medium-chain/long-chain mono-, di-, and triglycerides, glyceryl monocaprylate, and water-soluble cosolvents with hydrogenated castor oil constructing ternary phase diagrams for propofol. The developed SNEDDS formulations were characterized using visual observation, particle size analysis, zeta potential, transmission electron microscopy, equilibrium solubility, in vitro dynamic dispersion and stability, and in vivo sleeping disorder studies in rats. The in vivo bioavailability of the SNEDDSs in rats was also studied to compare the representative formulations with the marketed product Diprivan^®^. Results: Medium-chain triglycerides (M810) with mono-diglycerides (CMCM) as an oil blend and hydrogenated castor oil (KHS15) as a surfactant were selected as key ingredients in ternary phase diagram studies. The nanoemulsifying regions were identified from the studies and a number of SNEDDSs were formulated. Results from the characterization studies demonstrated the formation of efficient nanosized particles (28–45 nm globule size, 0.10–0.20 PDI) in the optimized SNEDDS with a drug loading of 50 mg/g, which is almost 500-fold higher than free propofol. TEM analysis showed the formation of spherical and homogeneous nanoparticles of less than 50 nm. The dissolution rate of the representative SNEDDS was faster than raw propofol and able to maintain 99% propofol in aqueous solution for around 24 h. The optimized liquid SNEDDS formulation was found to be thermodynamically stable. The intravenous administration of the SNEDDS in male Wistar rats induced a sleeping time of 73–88 min. The mean plasma concentrations after the IV administration of propofol nano-formulations PF2-SNEDDS and PF8-SNEDDS were 1348.07 ± 27.31 and 1138.66 ± 44.97 µg/mL, as compared to 891.44 ± 26.05 µg/mL (*p* = 0.05) observed after the IV administration of raw propofol. Conclusion: Propofol-loaded SNEDDS formulations could be a potential pharmaceutical product with improved stability, bioavailability, and anesthetic activity.

## 1. Introduction

Propofol is a relatively short-acting anesthetic drug that slows down the function of the brain and nervous system during surgery and other medical procedures [1,2]. The poor water solubility of propofol has pushed many marketed and clinical formulations to be oil-in-water (o/w) emulsion systems [1,3]. Propofol belongs to the group of BCS class II drugs regarded as having low solubility (solubility in water 0.124 mg/mL) and high permeability [3]. Therefore, an insufficient concentration of the drug reaches the systemic circulation, leading to poor therapeutic efficacy and patient compliance. Therefore, no oral dosage form is currently available, which usually requires the drug to be dissolved and released into the gastrointestinal fluid before absorption [4,5]. Most patients regain consciousness within 5 to 18 min after the infusion of propofol. For general anesthesia induction, the initial dose of propofol is 2 mg/kg and is given as 40 mg IV every 10 s until the onset of anesthesia. To maintain the induced anesthesia, the drug is infused at 0.1 mg/kg/min IV, usually for 3 to 5 min [6]. It can be run through a central or peripheral IV line and can be paired with fentanyl for pain relief. However, a slow infusion is preferred over rapid bolus administration. It should not be administered through the same IV catheter with blood or plasma to decrease the pain associated with injection; the larger veins of the forearms can be considered [7]. The drug is now recommended for non-anesthetic procedures such as those in the emergency room and treating difficult migraine and non-migraine headaches [8]. The lipophilic nature of propofol accounted for the initial formulation development with Cremophor EL (CrEL) for human use [8]. However, there were numerous side effects caused by CrEL e.g., severe pain on injection as well as severe anaphylactoid reactions that compromised the safe use of propofol [9]. Improvements in formulation approach introduced 1% of propofol in a 10% soybean oil emulsion as a marketed product (Diprivan^®^ Injectable Emulsion by AstraZeneca) to avoid the CrEL-induced anaphylaxis, which still showed significant side effects (pain on injection), hyperlipidemia (elevated triglycerides and propofol infusion syndrome), and bacterial growth but were outweighed against propofol’s superior anesthetic properties compared to already-existing anesthetics [10]. It is generally believed that the small quantity of propofol in the aqueous phase of the 10% soybean oil emulsions is responsible for undesirable injection pain. A further-modified emulsion (Ampofol^®^—1% propofol emulsion containing 5% soybean oil and 0.6% lecithin) with protein, an improved formulation with fewer side effects due to the low-oil-containing emulsion, was less supportive of microbe growth because of the higher propofol-to-lipid ratio [11]. One of the strategies to avoid these side effects was the optimization of the surfactant or using a different emulsifying agent in the formulation [12]. However, the o/w emulsion formulations do not have adverse allergic reactions and also the addition of EDTA to the emulsion offers antimicrobial properties, lowering the incidence of sepsis [13]. The significant rise in the demand for minor invasive surgical procedures accounts for the increased global use of propofol. The market share size for propofol in the United States increased from $116 million in the year 2010 to $246 million in 2013, and the share has been rising exponentially over the years since it is the most used parenteral anesthesia in the United States.

Although several drug delivery systems such as crystallization, particle size reduction, inclusion complexes, solid lipid nanoparticles, solid dispersions, and nanocrystals have been developed with the objective to increase solubility and dissolution, due to various limitations (e.g., morphology changes, organic solvent usage, tedious, cost, etc.), they are pharmaceutically and therapeutically ineffective [14]. Therefore, a suitable drug delivery system that provides high therapeutic efficacy with fewer side effects is needed. Currently, the self-nanoemulsifying drug delivery system (SNEDDS) has been one of the growing strategies in academics as well as in pharmaceutical industries for improving the therapeutic efficacy of drugs by maintaining the drug in solubilized form throughout the gastrointestinal tract [15]. The basic components of the self-nanoemulsifying drug delivery system are safe and biocompatible and approved by the Food and Drug Administration as “Generally regarded as safe” (GRAS) [16]. SNEDDSs are basically an isotropic mixture of oil, surfactant, and cosolvent, which spontaneously form nano-sized droplets (SNEDDS) when in contact with gastrointestinal fluids upon mild agitation [17]. Nano-droplets have a larger surface area along with enhanced drug solubility and an adequate dissolution rate, thereby promoting drug absorption and leading to increased oral bioavailability [4]. The current research discusses the design and characterization of novel SNEDDS development for intravenous (iv) administration, which would be able to provide superior self-emulsification efficiency with improved physical stability and high drug-loading capacity. However, with the propofol oral dosage form, the sleeping disorder studies in vivo (rat model) had a very minimal effect (our unpublished data), and therefore it was not recommended to continue further oral pharmacokinetic investigations. Instead, the SNEDDS formulations were redesigned and administered intravenously and/or intraperitoneally for sleeping disorder and pharmacokinetic evaluation. The results were very promising compared to the raw drug and marketed product. We strongly believe that the developed intravenous SNEDDS formulations have clinical potential to improve the efficacy of propofol as dosage form.

## 2. Materials and Methods

### 2.1. Materials

Propofol (purity 99.9%) was obtained from Incepta Pharmaceuticals Ltd. (Dhaka, Bangladesh). Zanthoxylum rhetsa seed oil (ZRO) was collected by a steam distillation process from naturally obtained seeds (no organic solvent was used). Medium-chain triglycerides (carbon chain length 8–12), Miglyol 810 (M810), and corn oil (CO) were purchased from Sasol Germany GmbH (Werk, Witten, Germany). Capmul MCM (C10) mono/diglycerides of capric acid were purchased from Abitec Corp. Janesville, USA. Maisine 35–1 (long-chain monoglycerides) was obtained from Gattefosse SAS, (Saint-Priest, France). Kolliphor ELP and Kolliphor HS15 were purchased from BASF, Ludwigshafen, Germany. Tween 80 (polyoxyethylene sorbitan monooleate) was obtained from Sigma Aldrich, USA. HPLC-grade acetonitrile, methanol, formic acid, and phosphoric acid were obtained from BDH Chemicals Ltd., Poole, UK. Milli-Q water was obtained through a Milli-Q Integral Water Purification System (Millipore, Bedford, MA). All other reagents were of analytical grade and used without further purification.

### 2.2. Methods

#### 2.2.1. Animals

The male Wistar rats used in the current study were issued from the Central Animal House Facility of the College of Pharmacy, King Saud University (KSU), Riyadh, Saudi Arabia. The animals were kept at a constant temperature (24–25 °C) and humidity (60%) and provided with standard food and water.

#### 2.2.2. Development of Self-Nanoemulsifying Formulation

A series of self-nanoemulsifying formulations were prepared with 35% triglyceride oil, 15% mono- and diglyceride oil, and 50% surfactant to optimize the best suitable self-nanoemulsifying systems (SNEDDSs), which were investigated more closely for their characteristic features and utilization. Three different triglyceride oils and non-ionic surfactants were used to develop eight formulations. The optimized formulations were developed in anhydrous form and incorporated with the drug for maximum solubility.

#### 2.2.3. Pseudo-Ternary Phase Diagram Study

The optimization was carried out using an experimental design (Design Expert^®^, Stat-Ease, Inc., Minneapolis, MN, USA) to ensure the time-effective and accurate optimization of the formulations [18]. The optimized liquid SNEDDSs should be balanced between high drug solubility, acceptable self-emulsification efficiency, lower droplet size, and enhanced in vitro dispersion [19]. Ternary phase diagrams were constructed using three components representing lipid formulations at various stages of dilution. The primary mixture of 10 g was prepared by weighing different proportions of Miglyol 810 and Capmul MCM (oils), Kolliphor HS15 (surfactant), and/or water (cosolvent). The compositions were thoroughly mixed with a vortex mixer (VWR, Scientific Industries, Inc., Bohemia, NY, USA) and heated (<40 °C) to ensure homogeneity. The anhydrous mixtures were subsequently diluted with water at different percentages (10%, 20%, up to 90%). Samples were stored in glass tubes (12 × 100 mm Pyrex, lined screw cap) with watertight closures and equilibrated for 48 h at room temperature and at 37 °C in a water bath (Memmert, Germany) to reverse any phase changes that might have been induced by heating during mixing. Mixture compositions of the excipients are expressed as % (*w*/*w*).

Phase behavior was initially assessed by visual observation, classifying mixtures as single-phase or multiphasic (turbid) mixtures. Liquid crystalline (LC) phases were identified both at ambient temperature and at 37 °C using a polarizing plate (Cole-Parmer, USA) fitted with cross-polarizing filters. After the identification of the nanoemulsifying region in the phase diagrams, the efficient self-nanoemulsifying formulations were differentiated on the basis of their characteristic dilution profiles.

#### 2.2.4. Self-Emulsification Assessment

A previously reported [19,20] visual test for the evaluation of self-emulsification efficiency was modified and adopted for the current self-emulsification assessment study. Drug-loaded formulations were subjected to 1:1000 aqueous dilutions in a 50.0 mL glass beaker and then constantly mixed at ~500 rpm using a magnetic stirrer. The formulations were assessed in terms of excipient miscibility, spontaneity, and homogeneity/dispersibility as performance indicators. The self-assessment test was conducted at room temperature (22 °C) and the passing formulations should rapidly disperse within a minute or less.

#### 2.2.5. Particle Size, PDI, and Zeta Potential Analysis

The particle size distribution and zeta potential (to determine the stability of the formulation) of the diluted dispersed formulations were analyzed using a particle-size analyzer by Brookhaven (Model 90 plus, Particle Sizing unit). All self-emulsifying formulations were diluted at a ratio of 1:1000 *v*/*v* (formulation: Milli-Q water) and mixed for 1 min before analysis.

#### 2.2.6. Transmission Electron Microscopy (TEM)

Bright-field transmission electronic images of the liquid SNEDDSs were taken using JEOL, JSM-3010 TEM, Japan, which were operated at 300 keV. Samples for TEM measurements were prepared in a one-in-ten dilution with water and the solution of nanoparticles on a copper grid was supported by Formvar Films.

#### 2.2.7. Drug Loading with Propofol

The solubility of propofol within the self-emulsifying formulations was determined using the shake flask method [4]. Samples were prepared by adding an excess amount of the drug (200 mg) to 1 g of the formulation, followed by subsequent shaking and agitation with the vortex mixer to ensure adequate mixing. Replicate sample tubes (three) for each formulation were incubated at 37 °C in a dry heat incubator until equilibrium was reached. The equilibrated samples were removed and centrifuged in 1.5 mL Eppendorf tubes to separate the excess solid drug from the dissolved drug. Then, an aliquot of the resulting supernatant was taken by weight for dilution in appropriate solvent systems before analysis. The drug solubility of the formulations was determined using ultra-high-performance liquid chromatography (UHPLC) systems.

#### 2.2.8. Dynamic Dispersion Study

Following our previously published method for dynamic dispersion studies, the propofol was dissolved at a concentration representing 80% (40 mg) of its equilibrium solubility in the relevant anhydrous formulation [21]. Then, 1 g of each formulation was diluted in 100 mL of water representing a concentration of 400 µg/mL. The dispersion was subsequently agitated and kept in a dry heat incubator at 37 °C for 48 h. During this 48 h period, the dispersions were assayed periodically by UHPLC to monitor precipitation.

One milliliter from each tube was withdrawn after 0, 0.5, 1, 2, 4, 8, 16, 24, 32, and 48 h and centrifuged for 5 min at 13,200 rpm. Then, an aliquot (100 µL) of the supernatant from the Eppendorf tube was taken for dilution with 10 mL of the appropriate solvent prior to assay by UHPLC. All experiments were carried out in triplicate.

#### 2.2.9. Storage Stability

The storage stabilities of the representative propofol-loaded F2 SNEDDS (PF2-SNEDDS) and F8 (PF8-SNEDDS) SNEDDS were evaluated at 4 °C and 37 °C for 6 months. At the predetermined time points, samples were collected for droplet sizing, zeta potential, and solubility studies to examine whether the characteristics of PF2 and PF8 SNEDDS formulations were altered after storage.

#### 2.2.10. Measurement of Propofol-Induced Sleeping Time in Male Wistar Rats (IV and IP Administration)

Healthy male Wistar rats (220–250 g, n = 6) were kept in standard plastic animal cages in groups of 10 animals each with a 12 h light and dark cycle at 25 ± 2 °C. The study was approved by the animal facilities guidelines from the Ethical Committee of the Experimental Animal Care Center, College of Pharmacy, King Saud University (Ethics Reference No: KSU-SE-21-14; Mar, 2020). All the rats were fed on standard rat chow and provided water ad libitum. They were acclimatized to laboratory conditions for a week prior to the experiments.

For intravenous (IV) administration, five groups of 6 rats (n = 6) each were selected, and for intraperitoneal (IP) administration, four groups of 6 rats (n = 6) each were selected. Group I received the vehicle (2 mL of saline); Group II received raw propofol. Group III and IV received PF2-SNEDDS and PF8-SNEDDS, respectively, and Group V received the marketed product Diprivan^®^ by IV administration. Similarly for IP administration, Group I received the vehicle (2 mL of saline); Group II received raw propofol. Group III received PF2-SNEDDS, and Group IV received the marketed product Diprivan^®^ suspended in saline. The animals of Groups II, III, IV, and V were treated with an equivalent dose of propofol (10 mg kg^−1^, intravenously). The sleeping time of the rats was measured as the time interval between the onset and the regaining of the righting reflex.

#### 2.2.11. Pharmacokinetic Studies

Fifteen healthy male Wistar rats (200 ± 20 g) were divided into three groups (6 in each group). The animals were maintained in accordance with the recommendations of the “Guide for the Care and Use of Laboratory Animals” of the center that approved the study. This study was carried out in accordance with the principles of the National Institute of Health (NIH) Guide for the Care and Use of Laboratory Animals (Publications No. 80–23; 1996). The pharmacokinetic study was approved by the animal facilities guidelines from the Ethical Committee of the Experimental Animal Care Center, College of Pharmacy, KSU (Ethics Reference No: KSU-SE-21-14; Mar, 2020). All animals were kept under standard laboratory conditions (23 ± 2 °C, humidity 60%, and 12 h light–dark cycle), with unrestricted access to granulated standard food and water.

Group I animals received propofol standard (raw drug). Group II and III animals received propofol nano-formulations PF2-SNEDDS and PF8-SNEDDS, respectively.

After overnight fasting, IV administration was performed using a tail vein of the rats at a dose of 10 mg/kg (for each drug formulation) with sterile disposable syringes and needles. Blood samples (0.3 mL) were drawn using a cannula at the tail vein at the following intervals and immediately before administration. For IV administration dosing, the sampling was carried out up to 240 min (i.e., 5, 15, 30, 60, 120, 180, and 240). The blood samples were collected in Eppendorf tubes containing 8 mg of disodium EDTA as an anticoagulant agent. Equal volumes of normal saline were injected through the cannula to replace the fluid lost. Plasma was separated by centrifugation in Eppendorf tubes at 5000× *g* for 10 min and stored at −80 °C until the quantitative analysis of propofol.

The concentration of propofol in plasma samples of the rats was determined as follows: 800 μL of methanol was added to a 200 µL aliquot of plasma. After vortex mixing for 3 min, the resultant mixture was centrifuged at 5000× *g* for 10 min, and the organic layer was transferred to a clean tube and dried under a light stream of nitrogen at 45 °C. The residue was re-dissolved in 200 μL of methanol and injected into the GC-MS column [22,23]. The pharmacokinetic parameters such as Cmax, T-max, and AUC were determined.

The pharmacokinetic parameters of raw propofol and propofol nano-formulations (PF2-SNEDDS and PF8-SNEDDS) were calculated using non-compartmental analysis. The calculated parameters were as follows: the apparent half-life, T1/2; maximum concentration in plasma, Cmax; area under the curve, AUC; the oral volume of distribution, Vd/F; and oral clearance, Cl/F. All pharmacokinetic analyses were performed using WinNonlin software (version 4.1, Pharsight Corporation, Palo Alto, CA, USA) and the results are expressed as the mean ± SD.

The experimental procedures were under the approval of the KSU Animal Ethical Committee and in compliance with the National Institute of Health (NIH) Guide for Care and Use of Laboratory Animals [24].

#### 2.2.12. Statistical Analysis

Prism^®^ pad software (San Diego, CA 92108, USA) was used for all the data analyses. One-way analysis of variance (ANOVA) followed by post hoc tests (LSD) was applied to compare the solubility and droplet size results. A value of *p* < 0.05 was considered significant throughout the study.

## 3. Results and Discussion

### 3.1. Development of Self-Nanoemulsifying Formulation (SNEDDS)

A series of self-emulsifying formulations were prepared with different ratios of oil and surfactant to optimize the best-suitable self-nanoemulsifying systems (SNEDDSs), which were investigated more closely for their characteristic features and utilization. The optimized formulation was developed in liquid form and incorporated with propofol for maximum solubility [25].

Table 1 shows eight formulations that were developed using various concentrations of oil blends with three specific water-soluble surfactants. Medium- and long-chain triglyceride oils such as zanthoxylum rhetsa seed oil (ZRO), Miglyol 810 (M810), and corn oil (CO) were blended with Capmul medium-chain monoglycerides (CMCM) and Maisine long-chain monoglycerides (M35-1), respectively. The first two formulations (F1 and F2) contained 35% M810, whereas formulations F3, F4, and F5 contained 35% ZRO and F6 35% CO with 15% CMCM, respectively. Similarly, F7 and F8 consisted of 35% ZRO with 15% M35-1. Non-ionic surfactant Kolliphor ELP (KELP), Kolliphor HS15 (KHS15), and Tween 80 (T80) were used at 50% constantly in all eight formulations (Table 1). The non-ionic surfactants Kolliphor ELP (KELP) and Kolliphor HS15 (KHS15) are highly purified, endotoxin-controlled, pharma-grade version excipients. They form clear solutions in water and are suitable for formulations containing extremely sensitive APIs, particularly for parenteral applications. Due to the high stabilizing effect of KHS15, as well as other beneficial characteristics, both the representative PF2 and PF8 SNEDDSs were developed with KHS15. In addition, propofol SNEDDSs produce a high concentration of free propofol in the aqueous phase and thus are prone to reducing excipient-related side effects.

### 3.2. Pseudo-Ternary Phase Diagram Study

Ternary phase diagrams were constructed at two temperatures (room temperature 22 °C and 37 °C) using three components representing lipid formulations at various stages of dilution. The primary mixture was prepared by blending different proportions of oils and surfactants. The formulation blends were thoroughly mixed with a vortex mixer (VWR, Scientific Industries, Inc., Bohemia, NY, USA) to ensure homogeneity. Samples were stored in glass tubes with watertight closures and equilibrated for 48 h at room temperature (22 °C) and at 37 °C in a water bath (Memmert, Germany) to reverse any phase changes.

**Figure 1:**
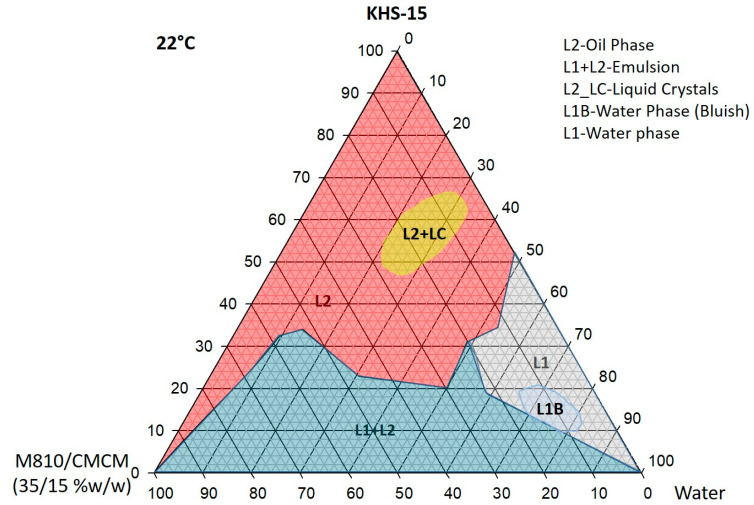
Ternary phase diagram of M810:CMCM (7:3)/HCO-30/water systems at 22 °C, in which L1 denotes a clear aqueous phase, L2 a transparent oily phase, L1 + L2 a milky emulsion, L2 + LC an oily phase with liquid crystals, and L1B a water continuous bluish phase.

**Figure 2:**
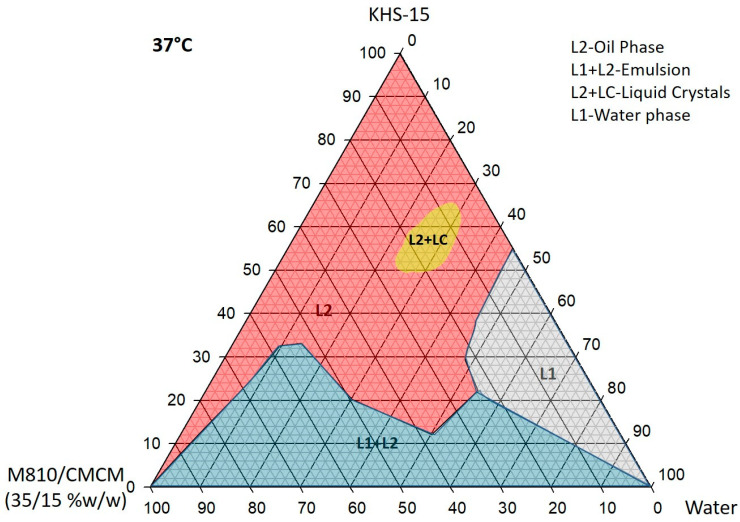
Ternary phase diagram of M810:CMCM (7:3)/HCO-30/water systems at 37 °C, in which L1 denotes a clear aqueous phase, L2 a transparent oily phase, L1 + L2 a milky emulsion, and L2 + LC an oily phase with liquid crystals.

Phase behavior was initially assessed by visual observation, classifying mixtures as single-phase or multiphasic (turbid) mixtures. Liquid crystalline (LC) phases were identified both at ambient temperature and at 37 °C using a polarizing plate (Cole-Parmer, USA) fitted with cross-polarizing filters. After the identification of the nanoemulsifying region in the phase diagrams, the efficient SNEDDS formulations were differentiated on the basis of their characteristic dilution profiles. L2 and L1 areas represented the oil continuous and water continuous phases, which were transparent in appearance. These regions were identified as SNEDDSs within the scope of the current studies. L1 + L2 was an emulsion region, milky in appearance, whereas L1B was a water continuous region with a bluish color phase. The F2—M810/CMCM/KHS15 (35/15/50 %*w*/*w*/*w*)—SNEDDS formulation was chosen from the L2 region of the phase diagram for further experimental studies (Figure 1 and Figure 2).

The ternary phase diagram has been widely used to identify the SNEDDS region and optimize the appropriate concentration of the oil, surfactant, and cosurfactant for designing the self-nanoemulsifying formulation. The low concentrations of surfactant KHS15 (0–30%) with an oil blend (M810/CMCM (7:3)) (100–70%) produced a milky emulsion (L1 + L2) with water within the phase diagram. After increasing the surfactant concentration at the expense of the oil, the system produced a large transparent (L2) phase by taking up 40–50% water. Within 20–25% water with 80–75% surfactant, the systems yielded a small LC area (transparent) in both phase diagrams. A clear aqueous phase (L1) was also prominent along the 55–95% water region with a small bluish region (L1B) separated at 80–85% water at 22 °C.

Upon increasing the temperature to 37 °C, the L2 phase within the ternary phase diagram was slightly expanded towards the L1 + L2 phase with the disappearance of the L1B (bluish) phase. This suggests that the miscibility was increased in systems (M810:CMCM (7:3)/KHS15) with water and can be expected to have better self-emulsification properties at stomach temperature (37 °C).

### 3.3. Self-Emulsification Assessment by Visual Observation

**Figure 3:**
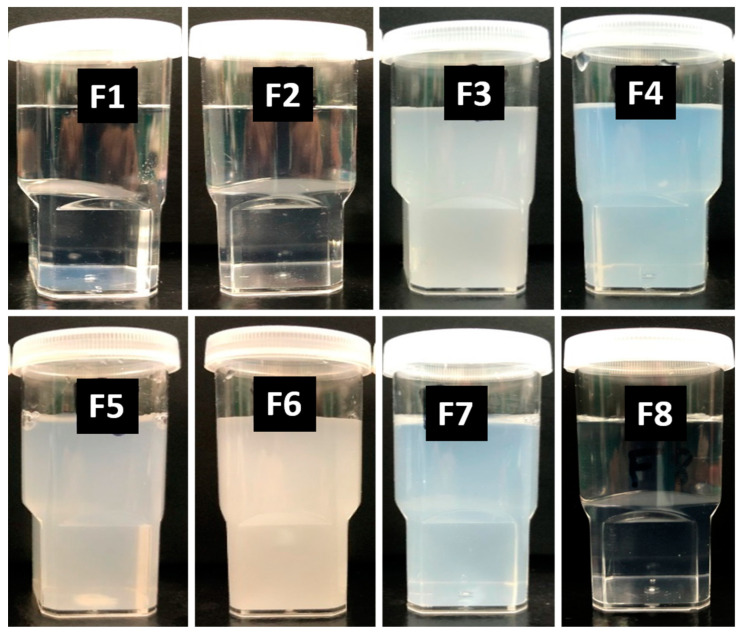
Appearances of the drug-free lipid-based formulations after aqueous dilution with water for the optimization of the representative SNEDDS. Formulations are F1: M810/CMCM/KELP (35/15/50); F2: M810/CMCM/KHS15 (35/15/50); F3: ZRO/CMCM/KELP (35/15/50); F4: ZRO/CMCM/KHS15 (35/15/50); F5: ZRO/CMCM/T80 (35/15/50); F6: CO/CMCM/KELP (35/15/50); F7: ZRO/M35-1/KELP (35/15/50); and F8: ZRO/M35-1/KHS15 (35/15/50) %*w*/*w*/*w*, respectively.

As previously reported [19,20] by using visual tests, the formulations were evaluated in terms of their excipient miscibility, appearance, and homogeneity/dispersibility (Figure 3).

By visual observation, it was easy to ensure uniform dispersibility, precipitation, and stability. The formulations were examined visually in terms of transparent/turbid/hazy/bluish appearance. All the formulations rapidly formed either within 1 min or less than a minute except for F6. The F6 anhydrous formulation was quite thick and produced a turbid appearance. The self-emulsification assessment images showed that only F1, F2, and F8 were compatible with surfactants, as they were homogeneous, transparent, and dispersed spontaneously (took less than a minute). Table 2 represents the self-emulsification efficiency results of eight formulations in anhydrous form and after their subsequent aqueous dilutions (1 in 1000) with water at room temperature (22 °C). The visual observation from the aqueous dilution of the formulations helped to optimize the representative SNEDDS for further studies.

### 3.4. Particle Size, PDI, and Zeta Potential Analysis

The particle size distribution, polydispersibility (PDI), and zeta potential of the diluted self-emulsifying formulations were measured successfully.

The droplet size analysis of the proposed SNEDDS formulations showed lower particle size distribution upon aqueous dilution. The formulations F1, F2, and F8 formed very fine dispersions with particle sizes of 28.08 nm, 28.81 nm, and 44.4 nm, respectively. Among these three formulations, the F2 system was found to be stable, with smaller particle size and higher zeta potential value upon aqueous dilution (Table 3). The data from the droplet size analysis suggested that more water-soluble excipients in the formulation lower the droplet size substantially upon aqueous dispersion. Hence, the droplet sizes of F1 and F2 were smaller than all other formulations due to the high contents of water-soluble excipients in the formulations. In addition, the F2-SNEDDS was widely monodispersed in the aqueous media with lower polydispersity values of less than 0.2.

The zeta potential value of F2 SNEDDS was -11.37 mV, as shown in Table 3. It was reported that the concentration of Kolliphor HS15 in the F2-SNEDDS sterically stabilized the system by forming a coating around its surface [26].

### 3.5. Transmission Electron Microscopy (TEM)

Bright-field transmission electronic images of the liquid SNEDDSs were taken using JEOL, JSM-3010 TEM, Japan, which was operated at 300 keV. TEM Samples were prepared in one-in-ten dilutions with water and the solution of the nanoparticles on a copper grid was supported by Formvar Films.

**Figure 4:**
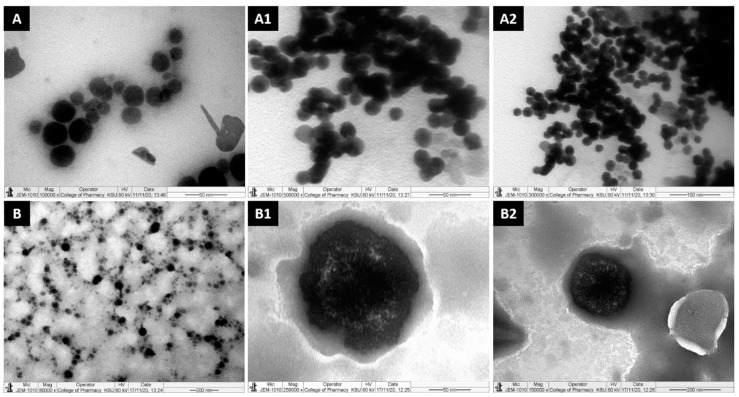
TEM micrographs of propofol-loaded representative formulations. (**A**,**A1**,**A2**) represent images of the droplet sizes of the PF2-SNEDDS at a 50–100 nm scale, and (**B**,**B1**,**B2**) represent images of the droplet sizes of the PF8-SNEDDS at a 50–200 nm scale.

TEM images of the propofol-loaded SNEDDS formulations following post-dilution with deionized water are shown in Figure 4. The TEM interpreted the surface morphology and globule/droplet size of the SNEDDS formulations. From the images (A, A1, A2), it was apparent that globules of the representative PF2-SNEDDS formulation were well dispersed and no globule aggregation took place, whereas the PF8-SNEDDS had shown (B, B1, B2) some aggregation and/or irregular size. The overall TEM data suggest that both the propofol-loaded PF2-SNEDDS and PF8-SNEDDS showed better spherical and homogeneous droplets and were in the nanosize range (30–50 nm), which was also evident by the particle size distribution data obtained using Brookhaven particle sizing systems (Table 3, see droplet size of F1 and F8 formulation).

### 3.6. Drug Loading and Maintaining Propofol in Solubilized Form

High drug solubilization is required for any drug to maintain the dose level with minimum excipient use. If the drug is orally given, it must be in a solubilized state in the stomach and small intestine for better absorption and systemic effects. In the current study, it was anticipated that SNEDDSs would improve the aqueous solubility of poorly water-soluble propofol. Therefore, the solubility of propofol was conducted in the self-emulsifying formulations using the shake flask method [4]. The drug solubility of the formulations was determined using GC/MS systems. From the current solubility experiment, it was confirmed that propofol was highly solubilized in the F1 and F8 nanoemulsifying formulations. Just 1 g of the anhydrous formulation was able to hold 50 mg propofol as a dosage form, which was double the strength of the marketed product (the marketed product is available at 25 mg/mL). Therefore, with our formulation, half of the excipients would be incorporated in the dosage form compared to the marketed product.

### 3.7. Propofol Loading into SNEDDS Formulation

The selection of SNEDDS formulations was performed based on their emulsification performances such as appearance, clarity, homogeneity, particle size, etc. for the model drug propofol. Appropriate solubility is the key factor influencing the effectiveness of the drug delivery system. Among the various oils, surfactants, and cosolvents, the maximum solubility of propofol was observed in the SNEDDSs containing medium- and long-chain triglycerides with mono- and diglycerides and the non-ionic surfactant Kolliphor HS15 (SNEDDS Formulation F2 and F8, Table 4). The maximum solubility of the drug in oil is an important factor that will otherwise lead to the precipitation of the drug and reduce its therapeutic efficacy. Miglyol 810 and ZRO are natural MCT/LCT oils used in lipid drug delivery systems as solubilizing agents. A surfactant is one of the basic components of a self-nanoemulsifying drug delivery system and especially non-ionic surfactants should be highly recommended because of their lower toxicity. Kolliphor HS15 is a highly hydrophilic non-ionic surfactant that is biologically compatible, non-toxic in nature, and less affected by pH and ionic strength. However, excess amounts may lead to allergic reactions or gastrointestinal irritation. An amount of 50 mg propofol was loaded in 1 g of each SNEDDS as a final dosage form (PF2-SNEDDS and F8-SNEDDS, Table 4). The drug solubility of the formulations was determined using GC-MS.

### 3.8. Method of GC-MS Analysis

GC-MS analysis was performed using a Perkin Elmer model Clarus 600 T system combined with a single quadrupole mass spectrometer. The samples were run at a 1 mL/min flow rate using an Elite 5MS chromatographic column (30 m × 0.25 mm × 0.25 µm film thickness) with high-purity helium as the carrier gas. The injector temperature was 280 °C and it was equipped with a splitless injector at 20:1. The initial temperature was set to 40 °C (held for 1 min), then further increased to 150 °C and 220 °C at 10 °C min^−1^ (held for 1 min), respectively. The MS ion source temperature was 220 °C and the inlet line temperature was maintained at 240 °C. The selected ion monitoring method was used to identify the propofol. The selected mass was 162.8 at 70 eV electron energy and a solvent delay of 7 min was applied.

**Figure 5:**
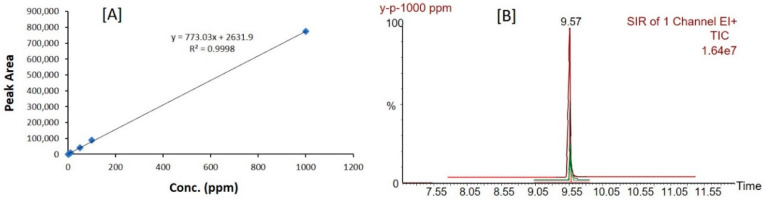
Calibration curve (**A**) and peaks (**B**) of propofol constructed by GC/MS sample analysis.

The peak response of propofol was linear over the concentration range between 1 and 1000 ppm (Figure 5A). Where *y* denotes: the peak area of the analyte and *x*: the concentration of the analyte (Figure 5A). These results show excellent linearity over the interval studied with correlation coefficient (r^2^) = 0.9998. The retention time of a single sample was 12 min (Figure 5B).

**Figure 6:**
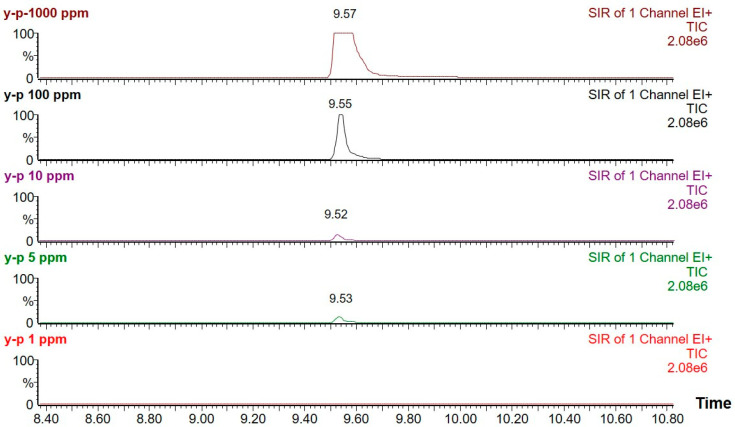
The chromatograms (TIC) of calibration standard (1000 ppm, 100 ppm, 10 ppm, 5 ppm, 1 ppm) using optimized GC-MS conditions.

The chromatographic results from the GC/MS technique suggested that, the separation of the propofol peak and its detection was ideal without any interference of the excipients used in SNEDDS dosage form. The propofol analyte was well separated at the retention time of ~9.57 min without having any interference of degradation product (Figure 6).

### 3.9. Dynamic Dispersion Study

The representative formulations that were investigated in equilibrium solubility studies after optimization were included in the current dynamic dispersion studies [27] to determine whether the drug was maintained in solubilized form after some time of dilution in aqueous media. By maintaining propofol in solution, the SNEDDS would able to show its stability and carry the maximum drug to the systemic circulation via absorption. Following a recent method for dynamic dispersion studies, the propofol was dissolved in each formulation at a concentration of 50 mg/g in the relevant anhydrous formulation. Then, 500 mg of each formulation was diluted in 50 mL water. The dispersion was subsequently agitated and kept in a dry heat incubator at 37 °C for 48 h. During this 48 h experimental period, the dispersion samples were assayed periodically by GC/MS to monitor precipitation.

One ml from each tube was withdrawn in an Eppendorf tube after 0, 0.5, 1, 2, 4, 8, 16, 24, 32, and 48 h and centrifuged for 5 min at 13,200 rpm for analysis by GC/MS. The data at 0–48 h intervals are provided in Table 5 showing the mass of propofol. The initial concentration was 100% propofol, which was dropped into the media. The concentration was reduced significantly with raw propofol (Table 5).

**Figure 7:**
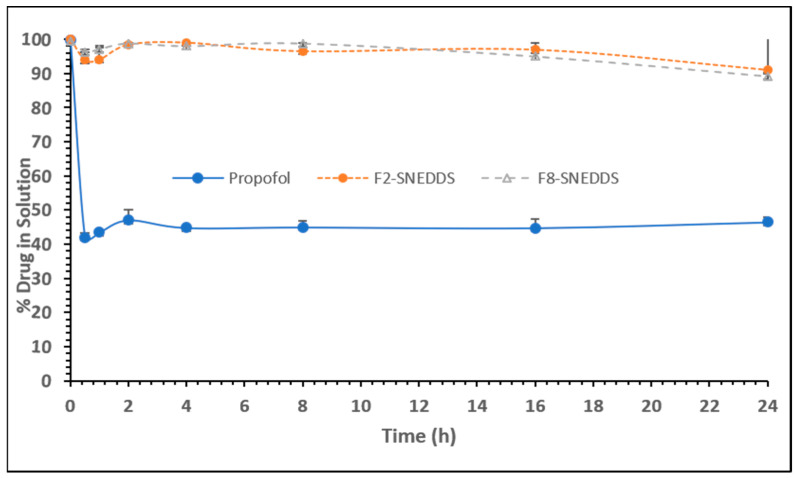
Percentage of propofol remaining in solution during 24 h after a dynamic dispersion study carried out with representative SNEDDS formulations. The formulations are F2-SNEDDS, M810/CMCM/KHS15 (35/15/50), and F8-SNEDDS, ZRO/M35-1/KHS15 (35/15/50) %*w*/*w*/*w*, respectively. Data are expressed as mean ± SD, n = 3.

The graph presented in Figure 7 indicates some differences in the precipitation of propofol from the PF2 and PF8 lipid-based SNEDDS formulations and propofol itself (raw drug). The aqueous dispersions of the PF2 (M810/CMCM/KHS15; 35/15/50%*w*/*w*/*w*) and PF8 (ZRO/M35-1/KHS15; 35/15/50%*w*/*w*/*w*) systems maintained approximately 99% of the drug in solution over the first 4 h period. This formulation would be expected to maintain propofol in solution for more than 24 h, which is long enough for the formulations to be exposed to the digestive system of the small intestine. This also suggests the stability of propofol in aqueous media at a maximum dilution level (1 in 100 dilution). In contrast, the free propofol resulted in extensive precipitation. These systems lost a significant proportion of the drug to precipitation and were able to maintain approximately 42% of the drug in solution. Therefore, raw propofol would not be suitable to use not only through an oral route but also intravenously. The precipitation behavior of the raw drug is more common due to the hydrophobic nature of the drug. However, if the drug is poorly soluble, the choice of the right lipid excipients is crucial for the successful delivery of the drug in a dosage form. In this case, a drug with high lipophilicity (log P value more than 3) could achieve a significant advantage in lipid-based SNEDDSs, which maintain the drug in solubilized form and improve stability. In our previous studies, it was confirmed that poorly soluble drugs precipitate depending on the formulation selection for the model drug under LFCS (lipid formulation classification systems) [28,29].

### 3.10. Stability Studies

Thermodynamic stability is the preliminary and most important parameter of a formulation to assess its stability. Thermodynamic stability was assessed on selected formulations from a phase diagram to identify and avoid metastability issues.

Thermodynamic stability is one of the main parameters to evaluate stability and help in the selection of an appropriate formulation. Particle size, dispersibility, and zeta potential were tested with and without loading the drug propofol. In our investigation, the particle size of the F2 SNEDDS was 28.81 nm without the drug, whereas it was found to be 34 nm after being loaded with the drug. Similarly, F8-SNEDDS produced 45.70 nm after loading, which was 44.40 nm before the drug loading (Table 6). Both the F2 and F8 SNEDDSs showed slight increases in particle size, which were insignificant. This suggests that propofol was not precipitated upon aqueous dilution and no phase separation occurred, either. No significant changes in the zeta potential and PDI values were observed in the propofol-free and propofol-loaded SNEDDS formulation at initial storage time and conditions (Table 6); however, the F2-SNEDDS showed a higher zeta potential of −16 mV with a propofol load. The higher zeta value suggests the improved stability of the propofol-loaded F2-SNEDDS formulation. In addition, the significant variation in the particle size of the F8-SNEDDS suggests a reduction in propofol after three months upon aqueous dispersion (Table 7).

On the other hand, the significance of the zeta potential value could be related to the SNEDDS stability. The dispersion of SNEDDSs with high zeta potential values either negative or positive are electrically stabilized. The negative zeta potential data show that particles are dispersed in a negative charge and vice versa [17]. From the data in Table 7, it is shown that both the PF2-SNEDDS and PF8-SNEDDS produced negative values at 4 °C and 37 °C. The zeta potential values of the PF2-SNEDDS were within −10 to −12 mV during 3 months of storage conditions, whereas the zeta values of the PF8-SNEDDS increased from −15.5 to −18.6 mV at 4 °C after 3 months of storage but decreased to −10.2 mV at 37 °C. This suggests that the stability of the PF8-SNEDDS might improve upon storage at 4 °C but could decrease at a high temperature such as 37 °C.

The representative F2 and F8 SNEDDS formulations were assessed for their physical stability at six months. In our study, no phase separation was observed during the storage of the formulations and also droplet size variations of the F2-SNEDDS were not observed after six months of storage at two different temperatures. On the other hand, the droplet size was significantly higher in the F8-SNEEDS formulations at 4 °C and 37 °C after six months (Table 8). The overall stability studies confirmed that F2 SNEDDS was able to retain its droplet size and zeta values at both temperatures.

**Figure 8:**
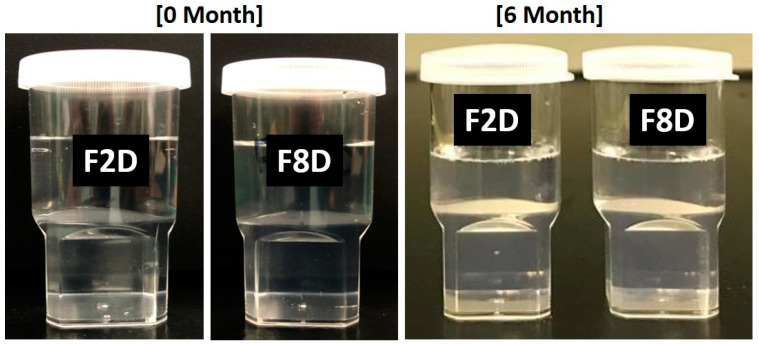
Appearances of the propofol-loaded F2 (PF2-SNEDDS) and F8 (PF8-SNEDDS) lipid-based formulations after six months of storage conditions at 37 °C. The formulations were diluted with water. “D denotes drug-loaded”. The left images represent at 0 months and the right images at 6 months.

Images of the physical stability test after six months are shown in Figure 8, carried out to estimate the maximum safe storage duration of the representative propofol SNEDDS. If the SNEDDS shows phase separation of the emulsion (cracking) phases and changes in its morphology upon aqueous dilution, it would be a loss of advantage. Hence, the test results showed no phase separation of both the F2 and F8 SNEDDS. In addition, no significant droplet size changes and zeta potential values at 37 °C were notable for the F2-SNEDDS, but there was an increase in droplet sizes was recorded for the F8-SNEEDS (droplet size analysis data shown in Table 8), The overall results suggested good physical stability in both the anhydrous and dispersed forms of the F2-SNEDDS.

### 3.11. Measurement of Propofol-Induced Sleeping Time in Male Wistar Rats (IV Administration)

**Figure 9:**
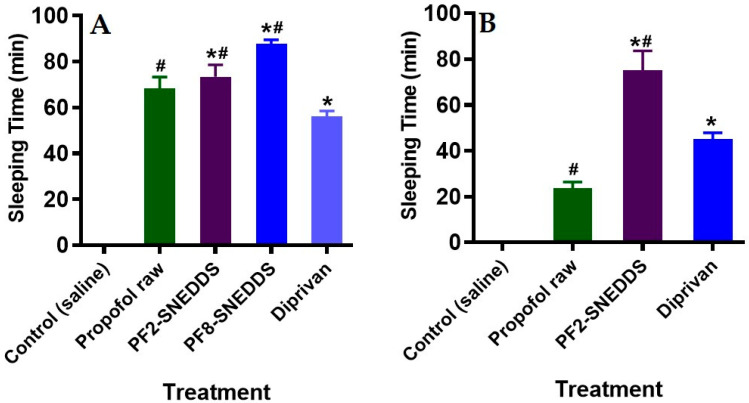
Measurement of propofol-induced sleeping time in rats (n = 6). (**A**) An equivalent dose of 10 mg/kg was administered intravenously to all the treatment groups (propofol raw, propofol-loaded formulation PF2-SNEDDS, PF8-SNEDDS, and marketed drug Diprivan^®^). (**B**) The dose (propofol raw, propofol-loaded formulation PF2-SNEDDS, and marketed drug Diprivan^®^) was administered intraperitoneally (IP). All results are presented as the average ± SEM. The asterisk and hash symbols (above the bars) indicate significant differences between samples * *p* < 0.05 (Propofol raw); # *p* < 0.05 (Diprivan^®^).

The data from the study in Figure 9A suggest that both the representative F2-SNEDDS and F8-SNEDDS formulations induced higher sleeping times of 73.33 min and 87.66 min, compared to raw propofol at 68.33 min (*p* < 0.05) and the marketed product Diprivan^®^ at 56 min (*p* < 0.05), respectively.

### 3.12. Measurement of Propofol-Induced Sleeping Time in Male Wistar Rats (IP Administration)

The data from the study in Figure 9B suggest that the representative PF2-SNEDDS formulation induced a higher sleeping time of 75 min compared to the raw propofol at 23.61 min (*p* < 0.05) and the marketed product Diprivan^®^ at 45 min (*p* < 0.05), respectively. Here, it can be seen that the raw propofol had very low performance on anesthetic effect compared to our developed PF2-SNEDDS. Similar performance was also noticed in the case of the marketed product, which was significantly low. This data suggests that due to the poor aqueous solubility of propofol, the raw propofol was not absorbed intraperitoneally. However, the representative PF2-SNEDDS could improve aqueous solubility and/or maintain the drug in solution (data shown in Figure 7), which leads to high propofol absorption to the systemic circulation.

The overall sleeping disorder studies in both IV and IP delivery systems (Figure 9) suggest that propofol SNEDDSs can be given to patients using both routes of administration. However, raw propofol is not suitable for IP administration, although it can be given intravenously, but it could have toxicity issues.

### 3.13. Propofol Bioavailability Studies

**Figure 10:**
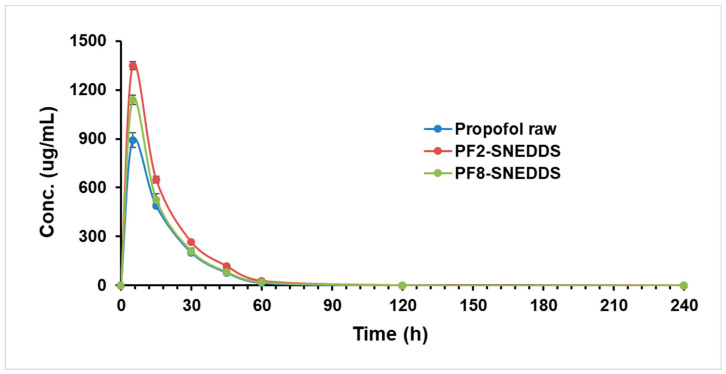
Plasma concentration–time profiles of propofol SNEDDS formulations PF2, PF8, and raw propofol (STD) after a single IV administration to rats at a dose equivalent to 10 mg/kg propofol (mean ± SEM, n = 6).

Drug formulations were well tolerated up to the target dose of 10 mg/kg (IV) by all animals. Plasma concentration–time curves of the drug and formulations in rats following IV administration are shown in Figure 10. The mean plasma concentration versus time curves after the IV administration of propofol nano-formulations (PF2-SNEDDS) and (PF8-SNEDDS) were 1348.07 ± 27.31 and 1138.66 ± 44.97 as compared to the standard raw propofol (891.44 ± 26.05 µg/mL) (*p* = 0.05). The systemic exposure, as measured by AUC_0-∞_, was 29,445.19 ± 1057.02 μg/mL*min and 24,140.19 ± 807.34 μg/mL*min, respectively, in animals receiving the PF2-SNEDDS and PF8-SNEDDS nano-formulations; this is in contrast to 20,354.33 ± 946.12 μg/mL*min in standard propofol rats (*p* = 0.05). The systemic T_1/2_ was increased to 10.12 ± 0.88 min and 9.71 ± 0.12 min in PF2-SNEDDS and PF8-SNEDDS group animals, respectively, from 9.74 ± 0.49 min in standard raw propofol animals (Table 9).

In the current studies, novel self-nanoemulsifying drug delivery systems (SNEDDS) were developed with lower droplet sizes and high zeta potential values (highly stable) for intravenous administration. The undesirable side effects of propofol for IV infusion could largely be associated with choices of surfactant and/or solvent [10]. One of the strategies to avoid these side effects was the optimization of the surfactant or using a different emulsifying agent in the SNEDDS formulation. Another reason for the pain on injection is probably due to the rapid release of propofol in the blood from the formulation. To minimize this side effect, the lipid composition of the SNEDDS formulation has been selected in terms of high propofol solubility (50 mg). Instead of using long-chain triglycerides, SNEDDS formulations were prepared with a blend of long- and medium-chain triglycerides/fatty acids, which could decrease the pain sensation on injection. By designing a new SNEDDS formulation with the use of another different surfactant (Kolliphor HS15), which leads to a more transient release of propofol, the pain on injection could theoretically be further diminished. However, this could lead to a slower release of propofol from the solvent and, hence, the slow onset of anesthesia [10].

Recently, a nanoemulsion formulation of propofol was prepared with semi-fluorinated surfactants [30,31]. These semi-fluorinated surfactants were previously used for intravenous drug delivery [32]. The formulations with different semi-fluorinated surfactants showed sedative properties equal to those of Diprivan^®^ and no signs of toxic effects and hence can be further developed and tested in clinical trials [31]. The research and developments are still ongoing to find the ideal vehicle for propofol which is nontoxic, not susceptible to microbial growth, stable, and has a low probability of inducing an allergic reaction. It should also maintain the optimal pharmacokinetic properties (quick onset, rapid recovery, and ideally should not cause pain on injection. These conditions make identifying the perfect formulation a real challenge.

## 4. Conclusions

The SNEDDS formulations were successfully prepared by GRAS-listed excipients, which can hold a high amount of propofol (50 mg per gram) and keep the drug in solution as a transparent nano-formulation (SNEDDS). The developed SNEDDS with enhanced stability can be given intravenously as a transparent liquid formulation (0.5 mg/mL). The current studies designed an efficient intravenous dosage form of propofol (liquid SNEDDS) with improved anesthetic activity, whereas the conventional formulation is turbid in appearance, has stability issues at room temperature, and causes pain during infusion. The proposed SNEDDS offers a promising delivery system for propofol which could be delivered at a maximum therapeutic dose intravenously depending on the patient’s needs.

## Figures and Tables

**Table 1:** The composition of the formulations developed for propofol. The systems show the composition of excipients used in each formulation at different ratios (%*w*/*w*).

F. No.	M810	ZRO	CO	CMCM	M35-1	KELP	KHS15	T80	Total%
F1	35			15		50			100
F2	35			15			50		100
F3		35		15		50			100
F4		35		15			50		100
F5		35		15				50	100
F6			35	15		50			100
F7		35			15	50			100
F8		35			15		50		100

**M 810.** Miglyol 810; ZRO: zanthoxylum rhetsa seed oil; CO: corn oil; CMCM: Capmul MCM; M35-1: Maisine 35-1; KELP: Kolliphor ELP; KHS15: Kolliphor HS15; T80: Tween 80; F-No.: denotes formulation number.

**Table 2:** Performance of lipid-based formulations on the basis of homogeneity, appearance, and self-emulsification capability.

Formulation	Compositions (%*w*/*w*)	Homogeneity	Appearance
F1	M810/CMCM/KELP (35/15/50)	Yes	Transparent
F2	M810/CMCM/KHS15 (35/15/50)	Yes	Transparent
F3	ZRO/CMCM/KELP (35/15/50)	Yes	Turbid
F4	ZRO/CMCM/KHS15 (35/15/50)	Yes	Bluish
F5	ZRO/CMCM/T80 (35/15/50)	Yes	Hazy
F6	CO/CMCM/KELP (35/15/50)	No	Turbid
F7	ZRO/M35-1/KELP (35/15/50)	Yes	Bluish
F8	ZRO/M35-1/KHS15 (35/15/50)	Yes	Transparent

**Table 3:** Mean particle size and PDI values of the different propofol-loaded liquid SNEDDS formulations. Data are expressed as mean ± SD, n = 3.

Formulation	Particle Size (nm)	PDI	Zeta Potential (mV)
F1	28.08 ± 0.21	0.089 ± 0.027	−4.10 ± 0.14
F2	28.81 ± 0.28	0.112± 0.014	−11.37 ± 0.70
F3	426.2 ± 11.0	0.71 ± 0.14	−29.2 ± 1.20
F4	128.0 ± 1.6	0.184 ± 0.011	−29.3 ± 0.60
F5	181.26 ± 1.67	0.253 ± 0.014	−28.4 ± 1.10
F6	526 ± 140	0.602 ± 0.017	−11.3 ± 0.50
F7	130.90 ± 0.70	0.258 ± 0.010	−25.5 ± 1.20
F8	44.4 ± 0.20	0.176 ± 0.008	−14.3 ± 0.70

**Table 4:** The propofol dissolved in the optimized formulations at 50 mg per gram of formulation.

Formulation	Compositions (%*w*/*w*)	Solubility (mg/g)
PF2-SNEDDS	M810/CMCM/KHS15 [35/15/50]	50
PF8-SNEDDS	ZRO/M35-1/KHS15 [35/15/50]	50

**Table 5:** Effect of dynamic dispersion on the solubilization profiles of propofol (initially 25 mg of propofol was dissolved in water). Data are expressed as mean ± SD, n = 3.

Time (h)	Drug Concentration (mg)		
	Propofol	PF2-SNEDDS	PF8-SNEDDS
0	24.97 ± 0.12	25.02 ± 0.56	24.87 ± 0.99
0.5	10.50 ± 0.28	23.49 ± 0.78	24.07 ± 0.00
1	10.88 ± 0.00	23.54 ± 0.84	24.29 ± 0.27
2	11.76 ± 0.75	24.61 ± 0.13	24.69 ± 2.28
4	11.20 ± 0.05	24.75 ± 1.32	24.50 ± 0.02
8	11.24 ± 0.49	24.13 ± 1.43	24.69 ± 0.06
16	11.19 ± 0.66	24.24 ± 0.13	23.74 ± 1.01
24	11.61 ± 0.35	22.78 ± 2.31	22.30 ± 0.21

**Table 6:** Mean particle size, PDI, and zeta potential values of the propofol (PF)-loaded SNEDDS and PF-free SNEDDS formulations at 0 time.

Formulation	Particle Size		Zeta Potential		PDI	
	Without PF	With PF	Without PF	With PF	Without PF	With PF
**F2**	28.81 ± 0.28	34.00 ± 0.27	−11.37 ± 0.70	−16 ± 1	0.112 ± 0.014	0.229 ± 0.005
**F8**	44.40 ± 0.20	45.70 ± 0.20	−14.3 ± 0.6	−15.5 ± 0.9	0.176 ± 0.008	0.198 ± 0.013

**Table 7:** Mean particle size, PDI, and zeta potential values of the PF-loaded SNEDDS formulations after 3 months of storage at 4 °C and 37 °C, respectively.

Formulations	4 °C		37 °C	
	F2	F8	F2	F8
**Particle size**	36.74 ± 0.1721	114.3 ± −2.68	40.9 ± 0.40	98.82 ± −0.31
**PDI**	0.184 ± −0.004	0.369 ± −0.014	0.260 ± −0.007	0.224 ± −0.004
**Zeta potential**	−10.4 ± −2.03	−18.6 ± −0.45	−12.2 ± −2.8	−10.2 ± −3.6

**Table 8:** Mean particle size, PDI, and zeta potential values of the PF-loaded SNEDDS formulations after 6 months of storage at 4 °C and 37 °C.

Formulations	4 °C		37 °C	
	F2	F8	F2	F8
**Particle size**	35.19 ± 0.101	85.35 ± −4.52	32.36 ± 0.32	97.13 ± 1.99
**PDI**	0.130 ± −0.009	0.206 ± −0.027	0.052 ± 0.009	0.261 ± 0.011
**Zeta potential**	−13.1 ± −3.15	−16.86 ± −1.44	−11.26 ± 0.45	−12.10 ± 1.51

**Table 9:** Comparison of pharmacokinetic parameters of propofol formulation F2-SNEDDS (PF2, M810/CMCM/KHS15; 35/15/50), PF8-SNEDDS (ZRO/M35-1/KHS15; 35/15/50) and raw propofol.

		Propofol Raw (STD)		PF2-SNEDDS		PF8-SNEDDS	
Parameter	Unit	Average	SD	Average	SD	Average	SD
Lambda_z	1/min	0.07	0.00	0.07	0.01	0.07	0.00
t1/2	min	9.74	0.49	10.12	0.88	9.71	0.12
Tmax	min	5.00	0.00	5.00	0.00	5.00	0.00
Cmax	μg/mL	891.44	26.05	1348.07	27.31	1138.66	44.97
AUC 0-t	μg/mL*min	20,130.69	885.63	29,038.38	879.13	23,866.09	800.63
AUC 0-inf_obs	μg/mL*min	20,354.33	946.12	29,445.19	1057.02	24,140.19	807.34
MRT 0-inf_obs	min	14.36	0.48	14.05	0.77	13.32	0.07
Vz_obs	(mg/kg)/(μg/mL)	0.01	0.00	0.00	0.00	0.01	0.00
Cl_obs	(mg/kg)/(μg/mL)/min	0.00	0.00	0.00	0.00	0.00	0.00
Vss_obs	(mg/kg)/(μg/mL)	0.01	0.00	0.00	0.00	0.01	0.00

T_max_ = time of maximum peak concentration; C_max_ = peak of maximum concentration; AUC_0→t_ = area under the concentration–time profile curve; AUC_0→∞_ = area under the concentration–time profile curve extrapolated to infinity; K_el_ = elimination rate constant, V_ss_ = volume of distribution at a steady state; T_1/2_ = half-life; MRT = mean residence time; CL/F = oral clearance.

## Data Availability

Not applicable.

## References

[1] Nilsson N., Nezvalova-Henriksen K., Tho I. (2019). Emulsion Stability of Different Intravenous Propofol Formulations in Simulated Co-Administration with Remifentanil Hydrochloride. Pharm. Technol. Hosp. Pharm..

[2] Niu K., Liu H., Chen R.W., Fang Q.W., Wen H., Guo S.M., Williams J.P., An J.X. (2018). Use of propofol for prevention of post-delivery nausea during cesarean section: A double-blind, randomized, placebo-controlled trial. J. Anesth..

[3] Wozniak K.M., Vornov J.J., Mistry B.M., Wu Y., Rais R., Slusher B.S. (2015). Gastrointestinal delivery of propofol from fospropofol: Its bioavailability and activity in rodents and human volunteers. J. Transl. Med..

[4] Mohsin K., Long M.A., Pouton C.W. (2009). Design of lipid-based formulations for oral administration of poorly water-soluble drugs: Precipitation of drug after dispersion of formulations in aqueous solution. J. Pharm. Sci..

[5] Buya A.B., Beloqui A., Memvanga P.B., Préat V. (2020). Self-Nano-Emulsifying Drug-Delivery Systems: From the Development to the Current Applications and Challenges in Oral Drug Delivery. Pharmaceutics.

[6] Lundström S., Twycross R., Mihalyo M., Wilcock A. (2010). Propofol. J. Pain Symptom Manag..

[7] Walsh C.T. (2018). Propofol: Milk of Amnesia. Cell.

[8] Uchegbu I.F., Jones M.-C., Corrente F., Godfrey L., Laghezza D., Carafa M., Holm P., Schatzlein A.G. (2014). The Oral and Intranasal Delivery of Propofol Using Chitosan Amphiphile Nanoparticles. Pharm. Nanotechnol..

[9] Baker M.T., Naguib M. (2005). Propofol: The challenges of formulation. Anesthesiology.

[10] Hulsman N., Hollmann M.W., Preckel B. (2018). Newer propofol, ketamine, and etomidate derivatives and delivery systems relevant to anesthesia practice. Best Pract. Res. Clin. Anaesthesiol..

[11] Le Guen M., Grassin-Delyle S., Cornet C., Genty A., Chazot T., Dardelle D., Liu N., Dreyfus J.F., Mazoit J.X., Devillier P. (2014). Comparison of the potency of different propofol formulations: A randomized, double-blind trial using closed-loop administration. Anesthesiology.

[12] Lee E.H., Lee S.H., Park D.Y., Ki K.H., Lee E.K., Lee D.H., Noh G.J. (2008). Physicochemical properties, pharmacokinetics, and pharmacodynamics of a reformulated microemulsion propofol in rats. Anesthesiology.

[13] Schläpfer M., Piegeler T., Dull R.O., Schwartz D.E., Mao M., Bonini M.G., Z’Graggen B.R., Beck-Schimmer B., Minshall R.D. (2015). Propofol increases morbidity and mortality in a rat model of sepsis. Crit. Care.

[14] Alshehri S., Imam S.S., Altamimi M.A., Hussain A., Shakeel F., Elzayat E., Mohsin K., Ibrahim M., Alanazi F. (2020). Enhanced dissolution of luteolin by solid dispersion prepared by different methods: Physicochemical characterization and antioxidant activity. ACS Omega.

[15] Mohsin K. (2003). Influence of Mesomorphic Structures on Solvent Capacity of Lipid Drug Delivery Systems. Ph.D. Thesis.

[16] Gibson L., Hauss D.J. (2007). Lipid-Based Excipients for Oral Drug Delivery. Oral Lipid-Based Formulations: Enhancing the Bioavailability of Poorly Water Soluble Drugs.

[17] Kazi M., Shahba A.A., Alrashoud S., Alwadei M., Sherif A.Y., Alanazi F.K. (2020). Bioactive Self-Nanoemulsifying Drug Delivery Systems (Bio-SNEDDS) for Combined Oral Delivery of Curcumin and Piperine. Molecules.

[18] Mukherjee T., Plakogiannis F.M. (2010). Development and oral bioavailability assessment of a supersaturated self-microemulsifying drug delivery system (SMEDDS) of albendazole. J. Pharm. Pharmacol..

[19] Shahba A.A.-W., Mohsin K., Alanazi F.K. (2012). Novel self-nanoemulsifying drug delivery systems (SNEDDS) for oral delivery of cinnarizine: Design, optimization, and in-vitro assessment. AAPS PharmSciTech.

[20] Shahba A.A.-W., Mohsin K., Alanazi F.K., Abdel-Rahman S.I. (2016). Optimization of Self-Nanoemulsifying Formulations for Weakly Basic Lipophilic Drugs: Role of Acidification and Experimental Design. Braz. J. Pharm. Sci..

[21] Mohsin K.A., Alanazi F. (2011). The fate of paclitaxel during in vitro dispersion testing of different lipid-based formulations. J. Drug Deliv. Sci. Technol..

[22] Weng T., Qi J., Lu Y., Wang K., Tian Z., Hu K., Yin Z., Wu W. (2014). The role of lipid-based nano delivery systems on oral bioavailability enhancement of fenofibrate, a BCS II drug: Comparison with fast-release formulations. J. Nanobiotechnol..

[23] Mohsin K., Alamri R., Ahmad A., Raish M., Alanazi F.K., Hussain M.D. (2016). Development of self-nanoemulsifying drug delivery systems for the enhancement of solubility and oral bioavailability of fenofibrate, a poorly water-soluble drug. Int. J. Nanomed..

[24] Shah N.H., Carvajal M.T., Patel C.I., Infeld M.H., Malick A.W. (1994). Self-Emulsifying Drug-Delivery Systems (Sedds) with Polyglycolyzed Glycerides for Improving in-Vitro Dissolution and Oral Absorption of Lipophilic Drugs. Int. J. Pharm..

[25] Kazi M., Alhajri A., Alshehri S.M., Elzayat E.M., Al Meanazel O.T., Shakeel F., Noman O., Altamimi M.A., Alanazi F.K. (2020). Enhancing oral bioavailability of apigenin using a bioactive self-nanoemulsifying drug delivery system (Bio-SNEDDS): In vitro, in vivo and stability evaluations. Pharmaceutics.

[26] Zhang H., Wang Z., Liu O. (2016). Simultaneous determination of kolliphor HS15 and miglyol 812 in microemulsion formulation by ultra-high performance liquid chromatography coupled with nano quantity analyte detector. J. Pharm. Anal..

[27] Kazi M., Al-Swairi M., Ahmad A., Raish M., Alanazi F.K., Badran M.M., Khan A.A., Alanazi A.M., Hussain M.D. (2019). Evaluation of self-nanoemulsifying drug delivery systems (SNEDDS) for poorly water-soluble talinolol: Preparation, in vitro and in vivo assessment. Front. Pharmacol..

[28] Kazi M., Al-Amri K.A., Alanazi F.K. (2017). The role of lipid-based drug delivery systems for enhancing solubility of highly selective antiviral agent acyclovir. Pharm. Dev. Technol..

[29] Devraj R., Williams H.D., Warren D.B., Mohsin K., Porter C.J.H., Pouton C.W. (2013). In vitro assessment of drug-free and fenofibrate-containing lipid formulations using dispersion and digestion testing gives detailed insights into the likely fate of formulations in the intestine. Eur. J. Pharm. Sci..

[30] Rittes J.C., Cagno G., Perez M.V., Mathias L.A. (2016). Comparative evaluation of propofol in nanoemulsion with solutol and soy lecithin for general anesthesia. Braz. J. Anesth..

[31] Deng T., Mao X., Li Y., Bo S., Yang Z., Jiang Z.X. (2018). Monodisperse oligoethylene glycols modified Propofol prodrugs. Bioorg. Med. Chem. Lett..

[32] Fast J.P., Perkins M.G., Pearce R.A., Mecozzi S. (2008). Fluoropolymer-based emulsions for the intravenous delivery of sevoflurane. Anesthesiology.

